# AUTHORS INDEX

**DOI:** 10.3400/avd.ai.20-01000

**Published:** 2020-12-25

## A

Abbassi, Omar Ahmed 28

Abe, Motoyuki 170

Abe, Satomi 86

Abiko, Akihiko 23

Acar, Altay Nihat 255

Aiba, Masahiro 351

Akai, Takafumi 52

Akasaka, Nobuyuki 355

Akkakrisee, Surasit 469

Aman, Aaiza 365

Aoki, Atsushi 240

Aoki, Hiroki 151

Aoki, Tomoyuki 343

Arima, Daisuke 90, 103

Arima, Takahiro 198

Asano, Naoki 430

Ashikaga, Takashi 370, 377

Azuma, Nobuyoshi 56, 86, 300

Azuma, Takashi 326

## B

Ban, Shinichi 430

Bolstad, Francesco 187

## C

Chiba, Kiyoshi 72, 269, 441

Chihara, Shingo 397

Chikada, Masahide 441

Chino, Shuji 180, 319, 330

Chiyoya, Mari 312

Chong, Tze Tec 390

Chua, Deborah 390

## D

Deguchi, Juno 107, 126

Diaz, Jos Antonio 1

Domoto, Satoru 326

## E

Egashira, Kensuke 4

Eguchi, Daihiko 235

Ekingen, Evren 255

Enta, Yusuke 194

## F

Fujii, Kosuke 76

Fujikawa, Tatsuya 109

Fujimori, Tomonari 45

Fujimoto, Daichi 187

Fujioka, Ayumu 13

Fukada, Joji 384

Fukuda, Ikuo 312, 437

Fukuda, Tetsuya 261

Fukuda, Wakako 437

Fukumoto, Yoshihiro 151

Fukutomi, Toshiaki 447

Furukawa, Tomokuni 137

Furusho, Aya 151

Furuyama, Tadashi 4

## G

Gon, Shigeyoshi 430

Guntani, Atsushi 404

## H

Hamaguchi, Shingo 176

Hamano, Kimikazu 410

Hara, Nobuhiro 370

Harada, Ryo 384

Harada, Takasuke 410

Hasegawa, Yuto 273

Hashimoto, Kazuki 176

Hashimoto, Kazunori 465

Hata, Masaki 194

Hayashi, Hiroyuki 170

Hayashi, Masanori 273

Henmi, Soichiro 316

Higashigawa, Takatoshi 180, 319

Hinooka, Ranko 86

Hirai, Hidekazu 418

Hirano, Koji 330

Hirano, Reina 330

Hiranuma, Wakiko 447

Hirata, Ken-ichi 291

Hirokawa, Masayuki 322

Hiromatsu, Shinichi 151

Hongsakul, Keerati 469

Honma, Kenichi 235

Hori, Daijiro 45, 163

Hori, Yusuke 13

Horiguchi, Sadaaki 461

Horii, Taiko 426

Horikawa, Takafumi 414

Horikawa, Yui 240

Hosaka, Norifumi 240

Hoshina, Katsuyuki 52

Hosoi, Kotaro 176

Huan, Khian Wan Sarah Joy 390

## I

Iba, Yutaka 183

Ichihashi, Shigeo 187

Ide, Toru 422

Igari, Kimihiro 144, 322

Iida, Osamu 56, 291, 300

Iio, Minami 69

Imaeda, Yusuke 198

Imamura, Yuki 312

Inage, Yuichi 96

Inoue, Ryosai 330

Inoue, Takehiro 76

Inoue, Takeshi 316

Inoue, Yosuke 261, 281

Inui, Tomohiko 96

Isaji, Toshihiko 52

Ishibashi, Hiroyuki 198

Ishida, Keiichi 96

Ishida, Masaru 404

Ito, Hidemichi 176

Ito, Hisato 330

Itoh, Takahito 69

## J

Jamil, Muhammad 365

Joseph, Feona Sibangun 347

## K

Kadotani, Makoto 291

Kadowaki, Tasuku 343

Kamada, Takeshi 384

Kamiya, Hiroyuki 355

Kamiya, Shinji 457

Kanamoto, Ryo 151

Kaneko, Mitsunori 437

Katayama, Keijiro 137

Kato, Hiroaki 180, 319

Kato, Noriyuki 180, 319

Kato, Otohime 248

Kato, Takayoshi 308

Katsui, Sotaro 144, 322

Kawaguchi, Yasuhiko 81

Kawaharada, Nobuyoshi 384

Kawai, Yujiro 69

Kawakubo, Eisuke 404

Kawamori, Hiroyuki 291

Kawamoto, Shunsuke 447

Kawasaki, Ryota 454

Kawase, Takumi 281

Kawashima, Motoharu 191, 454

Kawasugi, Kazuo 461

Kawatani, Yohei 339

Kichikawa, Kimihiko 187

Kigawa, Ikutaro 351

Kikuchi, Shinsuke 86

Kim, Taehwan 187

Kimura, Naoyuki 45, 163, 465

Kino, Shigeo 76

Kita, Shota 441

Kitahara, Hiroto 355

Kitamoto, Shohei 426

Kitamura, Hideki 81

Kitano, Ikuro 286

Kitayama, Hitoshi 76

Kiuchi, Ryuta 414

Kobayashi, Kimihiro 335

Koda, Yojiro 100

Kodama, Akio 56, 300

Koga, Jun-ichiro 4

Kohno, Hiroki 96

Koizumi, Kiyoshi 69

Komagamine, Masahide 441

Kondo, Norihiro 312

Konishi, Akihide 291

Kordzadeh, Ali 28

Kotoku, Akiyuki 72, 269

Koyama, Yutaka 81

Kozuki, Amane 187, 291

Kubota, Yuko 418

Kudo, Tomoaki 437

Kudo, Toshifumi 144, 322

Kudoh, Yasushi 444

Kunioka, Shingo 355

Kurahashi, Kanan 90, 103

Kuratani, Toru 422

Kurazumi, Hiroshi 410

Kurihara, Nobuhisa 322

Kurimoto, Yoshihiko 183, 384

Kuroda, Yoshinori 335

Kurosaki, Tatsuya 137

Kurose, Shun 4

Kusadokoro, Sho 163

## L

Lee, Tetsumin 370

Leong, Benjamin Dak Keung 347

## M

Maeda, Koichi 422

Maeda, Toshi 248

Maruhashi, Takaaki 72, 269

Maruta, Kazuto 240

Maruyama, Ryushi 183

Maruyama, Yuki 198

Masuda, Noriyasu 202

Masuda, Shinya 444

Masuda, Takahiko 183

Masuda, Tomoaki 240

Matoba, Satoaki 13

Matsubara, Shinobu 359

Matsuda, Hitoshi 100, 261, 281

Matsumiya, Goro 96

Matsumori, Masamichi 191

Matsumoto, Harunobu 45, 465

Matsumoto, Ryota 450

Matsumoto, Takuya 4

Matsunaga, Wataru 45

Matsuno, Yukihiro 434

Matsuo, Jiro 281

Matsuoka, Takayuki 447

Matsuura, Kaoru 96

Maze, Yasumi 330

Midorikawa, Hirofumi 343

Mii, Shinsuke 404

Mikami, Takuma 384

Mimura, Hidefumi 176, 269

Minagawa, Tadanori 447

Mishima, Akira 457

Mitsuoka, Hiroki 198

Mitsuoka, Hiroshi 273

Mitta, Shohei 434

Miura, Shuhei 183

Miyahara, Kazuhiro 52

Miyairi, Takeshi 72, 269, 441

Miyamoto, Takamichi 370

Miyashita, Naoya 76

Miyazaki, Ryoichi 370

Miyoshi, Kosuke 45

Mizoguchi, Takahiro 410

Moosa, Muhammad Asad 63

Mori, Masaki 4

Mori, Yoshiharu 457

Mori, Yoshio 434

Morikage, Noriyasu 410

Morisaki, Koichi 4

Morishita, Ryuichi 109

Moriya, Junji 176

Motoji, Yusuke 308

Mukohara, Nobuhiko 100, 191

Murakami, Hirohisa 100, 191, 454

Murakami, Kenji 441

## N

Naganuma, Masaaki 444

Nagase, Takashi 410

Nagata, Yasutoshi 370

Nagatomi, Satoru 187

Nagaya, Koichi 444

Nakagawa, Sayako 426

Nakai, Hidekazu 316

Nakai, Shingo 335

Nakai, Yosuke 457

Nakajima, Ken 180, 319

Nakamura, Bun 330

Nakamura, Hiromasa 343

Nakamura, Homare 176

Nakamura, Tomofumi 370

Nakano, Kaku 4

Naraoka, Syuichi 384

Natsume, Kayoko 273

Nawata, Kan 441

Nazir, Shahid 63

Nazir, Zafar 158

Niimi, Kazuho 430

Niinami, Hiroshi 326

Nishi, Satoshi 90, 103

Nishimaki, Hiroshi 72, 269, 441

Nishizawa, Masato 144, 322

Nomoto, Hidetsugu 377

Nomura, Yoshikatsu 191, 454

Nonaka, Takao 45, 465

Nozato, Toshihiro 370, 377

## O

Oba, Eiichi 335

Oba, Yasuhiro 81

Obara, Hideaki 273

Obayashi, Toru 370

Ochiai, Tomonori 335

Ogawa, Kaoru 312

Ogawa, Yukihisa 72, 176, 269, 441

Oguri, Atsushi 339

Ohara, Masato 447

Ohira, Seima 86, 355

Ohno, Masakazu 377

Ohno, Masato 437

Ohno, Nobuhisa 248

Ohta, Kengo 457

Ohta, Takashi 359

Ohtake, Hiroshi 414

Okada, Kenji 316

Okawa, Yasuhide 308

Okazaki, Jin 56, 404

Okazaki, Takanobu 137

Okita, Yutaka 108

Okugi, Satoshi 326

Omori, Takashi 291

Omoto, Tadashi 240

Omura, Atsushi 281, 316

Ono, Hisako 461

Ookura, Kazuhiro 273

Orimoto, Yuki 198

Ota, Kazunori 430

Ota, Tetsuo 86

Otsuka, Hiroyuki 151

Ouchi, Takafumi 180, 319

Oumi, Tetsuo 377

## P

Prionidis, Ioannis 28

## R

Rakugi, Hiromi 109

Rehman, Zia Ur 132, 158

Riaz, Amna 158

## S

Sadahiro, Mitsuaki 335

Saigan, Makoto 194

Saijo, Fumiyoshi 450

Saito, Masahito 430

Saito, Yoshiaki 312

Sakamoto, Kosuke 426

Sakuma, Hajime 180, 319

Sakurai, Yuka 72

Samura, Makoto 410

Sanada, Fumihiro 109

Sanada, Junichiro 414

Sasabuchi, Yusuke 45

Sasaki, Hiroaki 261, 281

Sato, Hirofumi 248

Sato, Hiroshi 384

Sato, Yasunori 273

Satoh, Yasuhiro 377

Sawa, Yoshiki 422, 450

Sawada, Kentaro 397

Seguchi, Ryuta 414

Seike, Yoshimasa 261, 281

Seki, Jun 308

Seo, Hiroyuki 418

Shahzad, Noman 63

Shaikh, Fareed Ahmad 63

Shawish, Emad 28

Shibata, Kana 109

Shibuya, Takashi 437, 450

Shijo, Takayuki 261

Shimamura, Kazuo 422

Shimizu, Haruna 137

Shimizu, Hideyuki 69

Shimizu, Shigeo 377

Shimizu, Takaharu 90, 103

Shimizu, Takuya 447

Shimizu, Toshikazu 163

Shimokawa, Hiroaki 116

Shindo, Tomohiko 116

Shinke, Toshiro 291

Shintani, Tsunehiro 273

Shintani, Yusuke 151

Shirasugi, Nozomu 461

Shirato, Hiroyuki 461

Shite, Junya 187

Shoji, Keisuke 13

Shomura, Yu 330

Sibata, Tsuyoshi 384

Siddiqui, Nadeem Ahmad 63

Skeik, Nedaa 38

Soga, Yoshimitsu 56, 300

Sophie, Ziad 63

Suda, Hisao 457

Suehiro, Kotaro 410

Suehiro, Shigefumi 418

Suehiro, Yasuo 418

Suematsu, Yoshihiro 90, 103

Sugimoto, Ikuo 300

Sugiyama, Tomoyo 377

Suzuki, Hirotoshi 441

Suzuki, Kenji 300

Suzuki, Masahito 377

Suzuki, Nobuaki 444

Suzuki, Ryo 410

Suzuki, Yoriyasu 81

## T

Tada, Norio 194

Tada, Yuki 355

Takagaki, Masami 343

Takahara, Mitsuyoshi 56, 300

Takahashi, Baku 457

Takahashi, Shinya 137

Takano, Hiroshi 430

Takao, Motoshi 330

Takasaki, Taiichi 137

Takayama, Toshio 52

Takeda, Miki 447

Takeuchi, Yuriko 410

Tamaki, Mototsugu 81

Tamiya, Yukihiko 384

Tamura, Yusaku 96

Tan, Chieh Suai 390

Tan, Ru Yu 390

Tanaka, Akiko 194

Tanaka, Hiroshi 454

Tanaka, Hiroyuki 151

Tanaka, Kiyoshi 404

Tanaka, Ryoichi 23

Tanaka, Shinichi 404

Tanaka, Takamitsu 461

Tang, Tjun Yip 390

Taniyama, Yoshiaki 109

Tatsukawa, Takamitsu 86

Terashi, Hiroto 56, 300

Thongkan, Tortrakoon 469

Tobinaga, Satoru 151

Tochikubo, Ai 86

Tokui, Toshiya 319, 330

Tomita, Shinji 308

Tomoeda, Hiroshi 397

Torikai, Kei 430

Tsuji, Yoriko 286

Tsuji, Yoshihiko 286

Tsukiyama, Yoshiro 291

Tsumura, Kosuke 308

## U

Uchida, Daiki 86

Uchida, Takaki 343

Uchida, Tetsuro 335

Ueda, Hideki 96

Uehara, Kyokun 261, 281

Ueno, Go 248

Ueno, Yousuke 343

Uetake, Hiroyuki 144, 322

Ujihira, Kosuke 183

Ukita, Rei 1

Umeda, Yukio 434

Usman, Rashid 365

Utsunomiya, Makoto 56

Uwabe, Kazuhiko 202

## W

Wada, Shinji 176

Watanabe, Go 414

Watanabe, Keita 370

Watanabe, Kenichi 437, 450

Watanabe, Michiko 96

Watanabe, Ryota 434

Westergard, Emily 38

## Y

Yakita, Yasunori 96

Yamada, Akira 183

Yamada, Tetsuya 198

Yamada, Toshiyuki 457

Yamada, Yu 422

Yamaguchi, Atsushi 45, 163, 465

Yamaguchi, Hiroki 343

Yamamoto, Kota 52

Yamanaka, Katsuhiro 316

Yamane, Yoshitaka 137

Yamasaki, Motoshige 339

Yamashita, Atsushi 335

Yamashita, Sho 4

Yamashita, Shu 377

Yamashita, Yoichi 426

Yamashita, Yoshiyuki 422

Yamauchi, Takashi 430

Yanaka, Kenichi 291

Yanase, Yohsuke 183

Yanishi, Kenji 13

Yap, Charyl Jia Qi 390

Yavuz, Kubra 255

Yavuz, Turhan 255

Yie, Kilsoo 93

Yokawa, Koki 261

Yokoi, Yoshihiko 326

Yokoyama, Nobu 45

Yoshiga, Ryosuke 404

Yoshimoto, Akihiro 90, 103

Yoshino, Shinichiro 4

Yoshioka, Kunihiro 23

Yoshizawa, Kosuke 248

Yukami, Shintaro 76

Yukawa, Arito 13

Yuri, Koichi 465

